# Region-adaptive magnetic resonance image enhancement for improving CNN-based segmentation of the prostate and prostatic zones

**DOI:** 10.1038/s41598-023-27671-8

**Published:** 2023-01-13

**Authors:** Dimitrios I. Zaridis, Eugenia Mylona, Nikolaos Tachos, Vasileios C. Pezoulas, Grigorios Grigoriadis, Nikos Tsiknakis, Kostas Marias, Manolis Tsiknakis, Dimitrios I. Fotiadis

**Affiliations:** 1 Biomedical Research Institute, Foundation for Research and Technology Hellas (FORTH), Ioannina, Greece; 2 Unit of Medical Technology and Intelligent Information Systems, Department of Materials Science and Engineering, University of Ioannina, Ioannina, Greece; 3 Institute of Computer Science, Foundation for Research and Technology Hellas (FORTH), Heraklion, Greece; 4 Department of Electrical and Computer Engineering, Hellenic Mediterranean University, Heraklion, Greece

**Keywords:** Prostate, Magnetic resonance imaging, Computer science

## Abstract

Automatic segmentation of the prostate of and the prostatic zones on MRI remains one of the most compelling research areas. While different image enhancement techniques are emerging as powerful tools for improving the performance of segmentation algorithms, their application still lacks consensus due to contrasting evidence regarding performance improvement and cross-model stability, further hampered by the inability to explain models’ predictions. Particularly, for prostate segmentation, the effectiveness of image enhancement on different Convolutional Neural Networks (CNN) remains largely unexplored. The present work introduces a novel image enhancement method, named RACLAHE, to enhance the performance of CNN models for segmenting the prostate’s gland and the prostatic zones. The improvement in performance and consistency across five CNN models (U-Net, U-Net++, U-Net3+, ResU-net and USE-NET) is compared against four popular image enhancement methods. Additionally, a methodology is proposed to explain, both quantitatively and qualitatively, the relation between saliency maps and ground truth probability maps. Overall, RACLAHE was the most consistent image enhancement algorithm in terms of performance improvement across CNN models with the mean increase in Dice Score ranging from 3 to 9% for the different prostatic regions, while achieving minimal inter-model variability. The integration of a feature driven methodology to explain the predictions after applying image enhancement methods, enables the development of a concrete, trustworthy automated pipeline for prostate segmentation on MR images.

## Introduction

Accurate segmentation of the prostate and the prostatic zones on T2w MRI consist the first step for a plethora of medical image analysis applications where clinically useful information needs to be extracted from the region of interest (ROI). Some of the most common applications are cancer detection and aggressiveness characterization, early prediction of recurrence, detection of metastases and assessment of treatment effectiveness, among others^1^. As the medical imaging domain moves toward sub-scale levels, with information being extracted from single voxels or pixels, segmentation accuracy is getting more demanding^2^. Particularly with the rise of radiomics analyses, any variability in the segmentation of the ROI will affect the numerical output of the features, thereby introducing bias into the evaluation of quantitative imaging biomarkers^3,4^. Furthermore, in the era of MRI-guided radiotherapy, precise organ and tumor delineation is of paramount importance as it may directly affect clinical outcomes^5,6^. Nevertheless, manual delineation of ROIs, not only is a time-consuming and labor-intensive task but also it thoroughly depends on the radiologist’s experience^7^.

To date, a plethora of Deep learning (DL) fully connected Convolutional Neural Network (CNN) pipelines have emerged to alleviate the burden of manual annotation in various radiological applications by automating and speeding up the segmentation process^8^. Most commonly, the backbone of such models is the U-net architecture^9^. There are a few comprehensive reviews on emerging DL applications for medical image segmentation^10–13^. Despite the state-of-the-art performance of novel architectures, the prostate and, particularly the prostatic zone segmentation, remains one of the most compelling research areas^14,15^.

Given the already large number of parameters included in state-of-the-art prostate segmentation models, image preprocessing, either by using denoising filters or contrast enhancement techniques, is aimed at increasing models performance by emphasizing key image characteristics relevant to the specific learning task^16^. An indispensable part of image preprocessing is image enhancement which aims to improve the visual quality of the image by modifying the intensity values of individual pixels so that anatomical structures can more easily be recognized by humans and machines. This is achieved by means of adequate gray-scale transformations^17^ aiming to disentangle the intensity distributions arising from adjacent regions with similar gray level intensities^18^. Therefore, by sharpening the boundaries between different tissues^19^, contrast enhancement has emerged as a powerful method for improving the accuracy of DL segmentation models^20^.

In the literature, several studies have demonstrated the effectiveness of image preprocessing to reduce the ambiguity of CNNs regarding their judgment and the feature extraction process^21^. There is a plethora of image enhancement techniques, many of which propose modifications of the Histogram Equalization algorithm (HE) or combinations of existing methodologies, such as the contrast limited adapted histogram equalization (CLAHE)^22^. The CLAHE algorithm consist one of the most popular and well-cited image enhancement techniques, as it appears to be particularly effective in medical imaging applications^23–25^. An improvement of CNN models’ performance on a variety of tasks has been reported after the application of image processing techniques, including object and texture classification^26,27^. This extends to medical imaging domain as well, in which, some authors have compared the effectiveness of image enhancement techniques for improving the quality of different imaging modalities (i.e. X-rays, CT, MRI) and for different clinical applications, such as lung, bone and vessel segmentation^23,28,29^, but also for disease detection and classification^30^. For instance, Rahman et al*.*^29^ evaluated the impact of various lung segmentation CNN algorithms and image enhancement techniques, including gamma correction, HE, CLAHE, image complement and Balance Contrast Enhancement Technique (BCET) on COVID-19 detection using X-ray images. The effect of image enhancement on liver segmentation from CT images, cervical cancer segmentation from T2W MR images, and vessel segmentation from 2D fundus images has also been investigated, suggesting that image enhancement prior to CNN model training leads to significant improvement in models’ performance^31–33^.

In this work, we propose an extension of the CLAHE method with the aim to improve the performance of state-of-the-art CNN models for segmenting the prostate gland and the prostatic zones. The performance of the proposed Region Adaptive CLAHE (RACLAHE) pipeline was compared against four prominent histogram-based image enhancement techniques, while the influence of the preprocessing methods on segmentation performance was assessed through the implementation of five well-established CNN models.

Overall, the main contributions of this study are the following:

- We propose an image enhancement method that consistently improves the performance of CNN segmentation models in T2 MR images of the prostate.
- We demonstrate, through feature map-driven visual explanations, that the proposed method is capable to enhance the image features that are most relevant to the segmentation task.
- We introduce a quantitative and qualitative feature importance metric to provide insights regarding DL segmentation models’ performance, thereby enhancing their explainability.
- To the best of our knowledge, this is the first study to quantitatively and qualitatively evaluate the effectiveness of image enhancement methods employing CNN models for prostate segmentation on MR images.

## Results

### Datasets description

The impact of four well-known histogram-based image enhancement methods along with the proposed region-adaptive technique were investigated for improving the segmentation of prostate’s whole gland (WG), transitional zone (TZ) and peripheral zone (PZ), using two publicly available datasets. One dataset was used for model training and another dataset was used to test externally the models’ performance. For model training, 204 patients from the Prostate-X dataset^34,35^ were used, along with the corresponding masks for the WG, TZ and PZ. The dataset consists of 3206 frames from Siemens' T2-weighted MR scans (TrioTim, Skyra models). For model testing, the Prostate 3-T^36^ dataset was employed, which included 30 patients and 421 frames with the associated annotations for all three regions acquired from Siemens' T2-weighted MRIs (Skyra model). In order to better examine the aforementioned prostatic areas, a descriptive analysis was conducted to quantify the inter- and intra-patient volume variations of the different prostatic regions (Supplementary Fig. S1).

### Evaluation of preprocessing methods

The metrics used for the evaluation of the proposed method were the Dice Score index (DS), the Rand Error Index (REI), the Sensitivity, the Balanced Accuracy (BA), the Hausdorff Distance (HD), and the Average Surface Distance (ASD). Tables 1, 2 and 3 show the prostate’s WG, the PZ and the TZ segmentation performance, respectively, of the five DL models using different image enhancement methods. For comparison, the models’ performance was also computed using the original images, without applying any enhancement. In the tables, it is also indicated whether the proposed RACLAHE performed significantly better than other preprocessing methods. The corresponding boxplots of DS, Sensitivity and HD are provided in Supplementary Figs. S2–S4 for the WG, PZ and TZ, respectively.

**Table 1:** Whole gland (WG) segmentation performance.

Model	Preprocessing method	Sensitivity	BA	DS	HD	ASD	REI
ResU-Net	W/o filter	0.63 ± 0.27*	0.81 ± 0.13*	0.72 ± 0.26*	10.4 ± 7.94*	3.38 ± 2.59*	0.21 ± 0.12*
	AGCWD	0.85 ± 0.19	0.92 ± 0.09	**0.85 ± 0.16**^†^	7.0 ± 6.67^†^	**1.96 ± 1.84**^†^	**0.14 ± 0.18**
	AGCCPF	0.72 ± 0.24*	0.86 ± 0.12*	0.78 ± 0.19*	9.88 ± 8.25*	2.97 ± 2.37*	0.2 ± 0.16*
	RLBHE	0.69 ± 0.28	0.84 ± 0.14	0.74 ± 0.25	10.65 ± 9.33	3.51 ± 3.63	0.2 ± 0.19
	CLAHE	**0.88 ± 0.18**^†^	**0.94 ± 0.09**^†^	0.85 ± 0.18^†^	**6.73 ± 6.45**^†^	2.02 ± 2.06^†^	0.14 ± 0.24*
	RACLAHE	0.85 ± 0.19	0.92 ± 0.09	0.83 ± 0.17	9.07 ± 9.46	2.55 ± 2.38	**0.14 ± 0.19**
U-Net	W/o filter	0.66 ± 0.23*	0.83 ± 0.11*	0.75 ± 0.21*	8.46 ± 6.62*	3.39 ± 3.11*	0.23 ± 0.13*
	AGCWD	0.78 ± 0.19*	0.89 ± 0.1*	0.83 ± 0.16	6.84 ± 5.77	2.32 ± 2.07	0.16 ± 0.1*
	AGCCPF	0.84 ± 0.16	**0.92 ± 0.08**	**0.86 ± 0.13**	**5.97 ± 5.28**^†^	**2.02 ± 1.76**^†^	0.16 ± 0.17
	RLBHE	0.69 ± 0.24*	0.85 ± 0.12*	0.76 ± 0.21*	8.2 ± 5.93*	3.08 ± 2.43*	0.2 ± 0.13*
	CLAHE	0.72 ± 0.24*	0.86 ± 0.12*	0.78 ± 0.2*	8.5 ± 6.29*	2.91 ± 2.5*	0.18 ± 0.1*
	RACLAHE	**0.85 ± 0.16**	**0.92 ± 0.08**	**0.85 ± 0.13**	6.99 ± 5.71	2.13 ± 1.62	**0.15 ± 0.17**
U-Net3+	W/o filter	0.83 ± 0.17	0.91 ± 0.08	0.85 ± 0.14	**6.18 ± 5.53**	**2.04 ± 1.62**	**0.15 ± 0.14**
	AGCWD	0.78 ± 0.2*	0.89 ± 0.1*	0.81 ± 0.17*	7.16 ± 4.96*	2.45 ± 1.58*	0.18 ± 0.14*
	AGCCPF	0.78 ± 0.21*	0.89 ± 0.11*	0.82 ± 0.18*	6.78 ± 4.95*	2.49 ± 1.9*	0.16 ± 0.13
	RLBHE	0.64 ± 0.23*	0.82 ± 0.11*	0.73 ± 0.2*	9.0 ± 6.28*	3.6 ± 2.48*	0.22 ± 0.1*
	CLAHE	0.69 ± 0.24*	0.85 ± 0.12*	0.76 ± 0.2*	8.94 ± 5.87*	3.14 ± 1.93*	0.2 ± 0.12*
	RACLAHE	**0.84 ± 0.17**	**0.92 ± 0.08**	**0.85 ± 0.14**	6.43 ± 5.93	2.28 ± 2.52	0.16 ± 0.18
U-Net++	W/o filter	0.77 ± 0.17*	0.88 ± 0.08*	0.81 ± 0.15*	6.83 ± 4.73*	2.68 ± 1.55*	0.19 ± 0.12*
	AGCWD	0.79 ± 0.17*	0.89 ± 0.09*	0.82 ± 0.15	**6.24 ± 4.47**	**2.48 ± 1.53**	**0.17 ± 0.12**☨
	AGCCPF	0.76 ± 0.21*	0.88 ± 0.1*	0.79 ± 0.17*	7.75 ± 5.75*	2.79 ± 1.95*	0.2 ± 0.16*
	RLBHE	0.75 ± 0.18*	0.87 ± 0.09*	0.8 ± 0.15*	6.82 ± 4.62*	2.75 ± 1.7*	0.2 ± 0.12*
	CLAHE	0.78 ± 0.21*	0.89 ± 0.1*	0.8 ± 0.18	7.57 ± 5.68*	2.57 ± 1.67	0.17 ± 0.15^†^
	RACLAHE	**0.86 ± 0.16**	**0.93 ± 0.08**	**0.83 ± 0.15**	6.78 ± 5.44	2.52 ± 2.0	0.18 ± 0.24
USE-NET	W/o filter	0.86 ± 0.15	0.93 ± 0.08	**0.88 ± 0.12**	**5.14 ± 4.62**	**1.82 ± 1.85**	**0.13 ± 0.16**
	AGCWD	0.86 ± 0.17*	0.93 ± 0.08*	0.87 ± 0.14^†^	5.54 ± 4.9^†^	1.9 ± 1.7^†^	0.13 ± 0.17
	AGCCPF	0.83 ± 0.16*	0.91 ± 0.08*	0.85 ± 0.14	6.11 ± 4.98	2.22 ± 2.12	0.15 ± 0.13^†^
	RLBHE	0.84 ± 0.15*	0.92 ± 0.08*	0.86 ± 0.12	5.92 ± 4.91	2.07 ± 1.91*	0.14 ± 0.14*
	CLAHE	0.83 ± 0.18*	0.91 ± 0.09*	0.85 ± 0.15	5.78 ± 4.99*	2.12 ± 2.22*	0.14 ± 0.14^†^
	RACLAHE	**0.89 ± 0.15**	**0.94 ± 0.07**	0.84 ± 0.15	6.6 ± 5.75	2.32 ± 2.19	0.16 ± 0.25

*BA* balanced accuracy, *DS* dice score, *HD* Hausdorff distance, *ASD* average surface distance, *REI* Rand error index, *AGCWD* adaptive gamma correction with weighting distribution, *AGCCPF* adaptive gamma correction with color preserving framework, *RLBHE* range limited Bi-histogram equalization, *CLAHE* contrast limited adaptive histogram equalization, *RACLAHE* region adaptive contrast limited adaptive histogram equalization.

*RACLAHE performs significantly better than the corresponding method (p-value ≤ 0.05; Wilcoxon rank-sum test).

^†^This method is significantly better than RACLAHE (p-value ≤ 0.05; Wilcoxon rank-sum test).

The best performance scores for each model are shown in bold.

**Table 2:** Peripheral zone (PZ) segmentation performance.

Model	Preprocessing method	Sensitivity	BA	DS	HD	ASD	REI
ResU-Net	W/o filter	0.67 ± 0.25*	0.83 ± 0.12*	0.68 ± 0.22	13.35 ± 15.73	2.54 ± 4.2	0.21 ± 0.13
	AGCWD	0.79 ± 0.22	0.89 ± 0.11	0.73 ± 0.19^†^	8.57 ± 7.77^†^	**1.7 ± 1.49**^†^	**0.15 ± 0.12**
	AGCCPF	**0.8 ± 0.17**	**0.9 ± 0.09**	**0.75 ± 0.17**^†^	9.38 ± 8.57^†^	1.77 ± 1.75^†^	0.17 ± 0.11
	RLBHE	0.67 ± 0.25*	0.83 ± 0.12*	0.68 ± 0.22	10.16 ± 8.81	2.16 ± 2.3	0.21 ± 0.13*
	CLAHE	0.67 ± 0.23*	0.83 ± 0.12*	0.71 ± 0.21	**9.31 ± 8.23**^†^	1.96 ± 2.18^†^	0.23 ± 0.13*
	RACLAHE	0.77 ± 0.22	0.88 ± 0.11	0.7 ± 0.19	11.39 ± 10.85	2.2 ± 1.9	0.16 ± 0.12
U-Net	W/o filter	0.61 ± 0.24*	0.8 ± 0.12*	0.66 ± 0.22*	**9.7 ± 9.11**	2.16 ± 2.35	0.23 ± 0.11
	AGCWD	0.58 ± 0.26*	0.79 ± 0.13*	0.63 ± 0.25*	10.3 ± 9.81	2.63 ± 3.67	0.25 ± 0.15*
	AGCCPF	0.58 ± 0.27*	0.79 ± 0.13*	0.64 ± 0.25	10.28 ± 8.84	2.3 ± 2.27	0.23 ± 0.13
	RLBHE	0.54 ± 0.26*	0.77 ± 0.13*	0.61 ± 0.25*	11.49 ± 9.4*	2.61 ± 2.79*	0.24 ± 0.11*
	CLAHE	0.48 ± 0.24*	0.74 ± 0.12*	0.57 ± 0.24*	11.46 ± 8.35*	2.69 ± 2.28*	0.25 ± 0.12*
	RACLAHE	**0.67 ± 0.22**	**0.83 ± 0.11**	**0.69 ± 0.21**	10.21 ± 9.5	**2.07 ± 1.92**	**0.22 ± 0.11**
U-Net3+	W/o filter	0.51 ± 0.25*	0.75 ± 0.12*	0.58 ± 0.24	11.16 ± 9.3	2.63 ± 2.38	0.25 ± 0.12
	AGCWD	0.49 ± 0.25*	0.75 ± 0.12*	0.57 ± 0.25	12.34 ± 10.26	3.07 ± 3.59*	0.27 ± 0.15
	AGCCPF	0.39 ± 0.24*	0.69 ± 0.12*	0.48 ± 0.25*	14.27 ± 10.45*	3.52 ± 2.87*	0.25 ± 0.15
	RLBHE	0.32 ± 0.23*	0.66 ± 0.11*	0.4 ± 0.24*	15.99 ± 10.45*	4.23 ± 3.11*	0.23 ± 0.15
	CLAHE	0.33 ± 0.24*	0.67 ± 0.12*	0.43 ± 0.26*	16.27 ± 9.46*	4.01 ± 2.75*	**0.22 ± 0.14**^†^
	RACLAHE	**0.57 ± 0.22**	**0.79 ± 0.11**	**0.62 ± 0.21**	**10.99 ± 9.47**	**2.47 ± 2.35**	0.26 ± 0.11
U-Net++	W/o filter	0.5 ± 0.23*	0.75 ± 0.11*	0.55 ± 0.22*	10.01 ± 8.8	2.59 ± 2.0	0.28 ± 0.14
	AGCWD	0.46 ± 0.21*	0.73 ± 0.1*	0.53 ± 0.2*	10.24 ± 8.47	2.57 ± 1.83*	0.27 ± 0.09*
	AGCCPF	0.54 ± 0.23*	0.77 ± 0.12*	0.57 ± 0.21*	10.19 ± 8.12	2.56 ± 1.88*	0.26 ± 0.12*
	RLBHE	0.49 ± 0.22*	0.74 ± 0.11*	0.54 ± 0.21*	10.99 ± 8.51*	2.79 ± 2.16*	0.27 ± 0.12*
	CLAHE	0.5 ± 0.22*	0.75 ± 0.11*	0.56 ± 0.22	10.18 ± 8.99	2.64 ± 2.59	0.29 ± 0.15*
	RACLAHE	**0.62 ± 0.21**	**0.81 ± 0.11**	**0.61 ± 0.2**	**9.88 ± 8.45**	**2.35 ± 1.76**	**0.25 ± 0.11**
USE-NET	W/o filter	0.63 ± 0.23*	0.82 ± 0.12*	0.69 ± 0.23	9.6 ± 8.09	1.94 ± 1.88	0.24 ± 0.12*
	AGCWD	0.64 ± 0.24*	0.82 ± 0.12*	0.68 ± 0.23	10.37 ± 8.49	2.3 ± 2.86	0.26 ± 0.17*
	AGCCPF	0.65 ± 0.24*	0.82 ± 0.12*	0.7 ± 0.23	9.69 ± 9.51	2.1 ± 2.79	0.25 ± 0.17*
	RLBHE	0.63 ± 0.24*	0.81 ± 0.12*	0.67 ± 0.23	9.91 ± 8.47	2.19 ± 2.42	0.25 ± 0.15*
	CLAHE	0.68 ± 0.21*	**0.84 ± 0.11**	**0.72 ± 0.21**^†^	**9.02 ± 8.74**	**1.85 ± 1.98**^†^	**0.22 ± 0.11**☨
	RACLAHE	**0.69 ± 0.25**	**0.84 ± 0.12**	0.68 ± 0.23	9.85 ± 9.11	2.23 ± 2.84	0.21 ± 0.16

*BA* balanced accuracy, *DS* dice score, *HD* Hausdorff distance, *ASD* average surface distance, *REI* Rand error index, *AGCWD* adaptive gamma correction with weighting distribution, *AGCCPF* adaptive gamma correction with color preserving framework, *RLBHE* range limited Bi-histogram equalization, *CLAHE* contrast limited adaptive histogram equalization, *RACLAHE* region adaptive contrast limited adaptive histogram equalization.

*RACLAHE performs significantly better than the corresponding method (p-value ≤ 0.05; Wilcoxon rank-sum test).

^†^This method is significantly better than RACLAHE (p-value ≤ 0.05; Wilcoxon rank-sum test).

The best performance scores for each model are shown in bold.

**Table 3:** Transitional zone (TZ) segmentation performance.

Model	Preprocessing method	Sensitivity	BA	DS	HD	ASD	REI
ResU-Net	W/o filter	**0.73 ± 0.25**^†^	0.86 ± 0.12*	0.76 ± 0.21*	11.43 ± 12.17*	3.01 ± 2.27*	0.2 ± 0.18
	AGCWD	0.58 ± 0.3*	0.79 ± 0.15*	0.66 ± 0.27*	10.24 ± 7.28*	3.43 ± 2.41*	0.22 ± 0.16*
	AGCCPF	0.63 ± 0.27*	0.81 ± 0.14*	0.7 ± 0.25*	9.59 ± 7.32*	3.38 ± 2.72*	0.23 ± 0.17*
	RLBHE	0.58 ± 0.29*	0.79 ± 0.14*	0.66 ± 0.26*	11.41 ± 9.38*	4.07 ± 3.4*	0.22 ± 0.14*
	CLAHE	0.72 ± 0.25	0.86 ± 0.13	0.76 ± 0.22	8.49 ± 8.11	2.73 ± 2.92	**0.19 ± 0.16**
	RACLAHE	0.72 ± 0.23	**0.86 ± 0.11**	**0.77 ± 0.19**	**7.4 ± 5.52**	**2.51 ± 1.87**	0.2 ± 0.13
U-Net	W/o filter	0.73 ± 0.23*	0.86 ± 0.12*	0.77 ± 0.19	**7.59 ± 6.23**	2.73 ± 2.32*	**0.19 ± 0.14**
	AGCWD	0.59 ± 0.27*	0.8 ± 0.14*	0.69 ± 0.25*	8.88 ± 6.65*	3.52 ± 2.73*	0.21 ± 0.11*
	AGCCPF	0.59 ± 0.25*	0.8 ± 0.13*	0.69 ± 0.23*	10.74 ± 7.19*	3.63 ± 2.38*	0.23 ± 0.11*
	RLBHE	0.63 ± 0.26*	0.82 ± 0.13*	0.72 ± 0.23*	8.62 ± 5.97*	3.21 ± 2.54*	0.22 ± 0.13*
	CLAHE	0.63 ± 0.27*	0.82 ± 0.13*	0.71 ± 0.23*	8.76 ± 6.79*	3.19 ± 2.59*	0.22 ± 0.14*
	RACLAHE	**0.76 ± 0.19**	**0.88 ± 0.09**	**0.8 ± 0.15**	7.73 ± 7.18	**2.54 ± 1.99**	0.2 ± 0.16
U-Net3+	W/o filter	0.63 ± 0.25*	0.82 ± 0.12*	0.71 ± 0.22*	8.62 ± 5.74*	3.2 ± 2.2*	0.22 ± 0.12*
	AGCWD	0.67 ± 0.23*	0.84 ± 0.11*	0.74 ± 0.2*	7.84 ± 5.07*	2.95 ± 1.8*	0.22 ± 0.12*
	AGCCPF	0.62 ± 0.27*	0.81 ± 0.13*	0.7 ± 0.24*	8.4 ± 5.18*	3.47 ± 2.45*	0.23 ± 0.15*
	RLBHE	0.42 ± 0.26*	0.71 ± 0.13*	0.53 ± 0.26*	12.54 ± 7.68*	5.87 ± 3.52*	0.23 ± 0.12*
	CLAHE	0.59 ± 0.26*	0.8 ± 0.13*	0.68 ± 0.24*	8.68 ± 5.43*	3.51 ± 2.4*	0.22 ± 0.12*
	RACLAHE	**0.79 ± 0.19**	**0.89 ± 0.09**	**0.8 ± 0.15**	**6.62 ± 4.5**	**2.39 ± 1.45**	**0.2 ± 0.19**
U-Net ++	W/o filter	0.67 ± 0.27*	0.84 ± 0.14*	0.72 ± 0.25*	8.13 ± 7.59*	3.23 ± 3.6*	**0.2 ± 0.15**☨
	AGCWD	0.64 ± 0.25*	0.82 ± 0.13*	0.71 ± 0.23*	8.01 ± 5.28*	3.42 ± 2.48*	0.22 ± 0.13*
	AGCCPF	0.41 ± 0.28*	0.71 ± 0.14*	0.51 ± 0.3*	14.54 ± 12.2*	5.85 ± 4.13*	0.21 ± 0.11*
	RLBHE	0.54 ± 0.28*	0.77 ± 0.14*	0.63 ± 0.26*	11.23 ± 8.39*	4.41 ± 3.04*	0.22 ± 0.12*
	CLAHE	0.6 ± 0.25*	0.8 ± 0.12*	0.69 ± 0.23*	9.04 ± 5.81*	3.86 ± 2.36*	0.23 ± 0.12*
	RACLAHE	**0.77 ± 0.21**	**0.88 ± 0.11**	**0.78 ± 0.17**	**6.64 ± 4.59**	**2.61 ± 1.53**	0.21 ± 0.2
USE-NET	W/o filter	0.68 ± 0.25*	0.84 ± 0.12*	0.76 ± 0.22*	7.36 ± 5.79*	2.82 ± 2.72	0.2 ± 0.11*
	AGCWD	0.71 ± 0.24*	0.85 ± 0.12*	0.77 ± 0.21	6.87 ± 5.61	2.65 ± 2.4	0.2 ± 0.15*
	AGCCPF	0.7 ± 0.24*	0.85 ± 0.12*	0.77 ± 0.2*	7.1 ± 5.49	2.79 ± 2.52	0.22 ± 0.17*
	RLBHE	0.73 ± 0.24*	0.86 ± 0.12*	0.78 ± 0.2	6.74 ± 4.99	2.51 ± 1.94	**0.19 ± 0.15**
	CLAHE	0.7 ± 0.24*	0.85 ± 0.12*	0.77 ± 0.2*	7.07 ± 5.68	2.66 ± 2.37	0.2 ± 0.14*
	RACLAHE	**0.8 ± 0.19**	**0.9 ± 0.09**	**0.81 ± 0.16**	**6.21 ± 4.03**	**2.31 ± 1.76**	0.2 ± 0.21

*BA* balanced accuracy, *DS* dice score, *HD* Hausdorff distance, *ASD* average surface distance, *REI* Rand error index, *AGCWD* adaptive gamma correction with weighting distribution, *AGCCPF* adaptive gamma correction with color preserving framework, *RLBHE* range limited Bi-histogram equalization, *CLAHE* contrast limited adaptive histogram equalization, *RACLAHE* region adaptive contrast limited adaptive histogram equalization.

*RACLAHE performs significantly better than the corresponding method (p-value ≤ 0.05; Wilcoxon rank-sum test).

^†^This method is significantly better than RACLAHE (p-value ≤ 0.05; Wilcoxon rank-sum test).

The best performance scores for each model are shown in bold.

Although, for WG segmentation the most robust networks tend to perform best without any image preprocessing (i.e. U-Net++, U-Net3+, USE-NET), the proposed RACLAHE algorithm was able to improve the sensitivity and BA in most cases, as shown in Table 1. AGCWD was efficient in improving U-Net and U-Net++ but degraded U-Net3+. With AGCCPF, only the performance of Unet was improved, achieving results similar to RACLAHE but degraded slightly U-Net++ , U-Net3+ and USE-NET. The CLAHE algorithm marginally outperforming RACLAHE for the ResU-net model, but degraded other networks such as U-Net3+. The RLBHE had the lowest performance compared to other methods with remarkably high variability. It is worth noting that the USE-NET model was the best performing network and remained invariant to image preprocessing. Even without any preprocessing, USE-NET achieved better scores for WG segmentation than all other models (i.e. AUC = 0.88 ± 0.12).

On the other hand, for the evaluation of prostate’s PZ segmentation, the RACLAHE algorithm consistently improved the performance of the majority of DL models, as it is shown in Table 2. The only exception was the ResU-net, for which AGCWD and AGCCPF achieved superior performance. Similar to WG segmentation task, for PZ segmentation, the models’ performance was degraded when the RLBHE was used. The CLAHE algorithm also degraded the models’ performance, except for USE-NET. Overall, the best performance was achieved with the ResU-net model (DS = 0.75 ± 0.17 for AGCCPF). Regarding the segmentation of prostate’s TZ, shown in Table 3, the proposed RACLAHE algorithm was the only consistent preprocessing method for significantly improving the performance of all the five networks. The best results for TZ segmentation were obtained for RACLAHE combined with the USE-NET model (DS = 0.81 ± 0.16). Overall, the average improvement across DL models in terms of DS was 3%, 8% and 9% for WG, PZ and TZ segmentation respectively.

### Inter-model performance and variability

The stability of each preprocessing method was computed in terms of method-specific average performance and variance in performance across all models. The inter-model performance, referring to the mean value for each method taking into consideration all the models, shows how each approach impacts DL segmentation models in general, and is described as follows:

1 $$Performance \left(m,filt\right)=mean\left({p}_{ResU-Net}\left(m,filt\right),{p}_{U-Net}\left(m,filt\right),{p}_{U-Net3+}\left(m,filt\right), {p}_{U-Net++}\left(m,filt\right),{p}_{USE-NET}\left(m,filt\right) \right),$$

where $m$ is the metric, $filt$ is the histogram processing methods, $p$ is the performance (mean score) for each model. This metric assists further for the identification of the best method as it reveals the method that has the most effective performance among models. In additions the inter-model variability shows how far are the mean values for each model from the $Performance (m,filt)$ evaluation metric. Typically represents the standard deviation across models ($std$) and it is reproduced by Eq. (2):

2 $$Variability \left(m,filt\right)=std\left({p}_{ResU-Net}\left(m,filt\right),{p}_{U-Net}\left(m,filt\right),{p}_{U-Net3+}\left(m,filt\right), {p}_{U-Net++}\left(m,filt\right),{p}_{USE-NET}\left(m,filt\right) \right).$$

**Figure 1:**
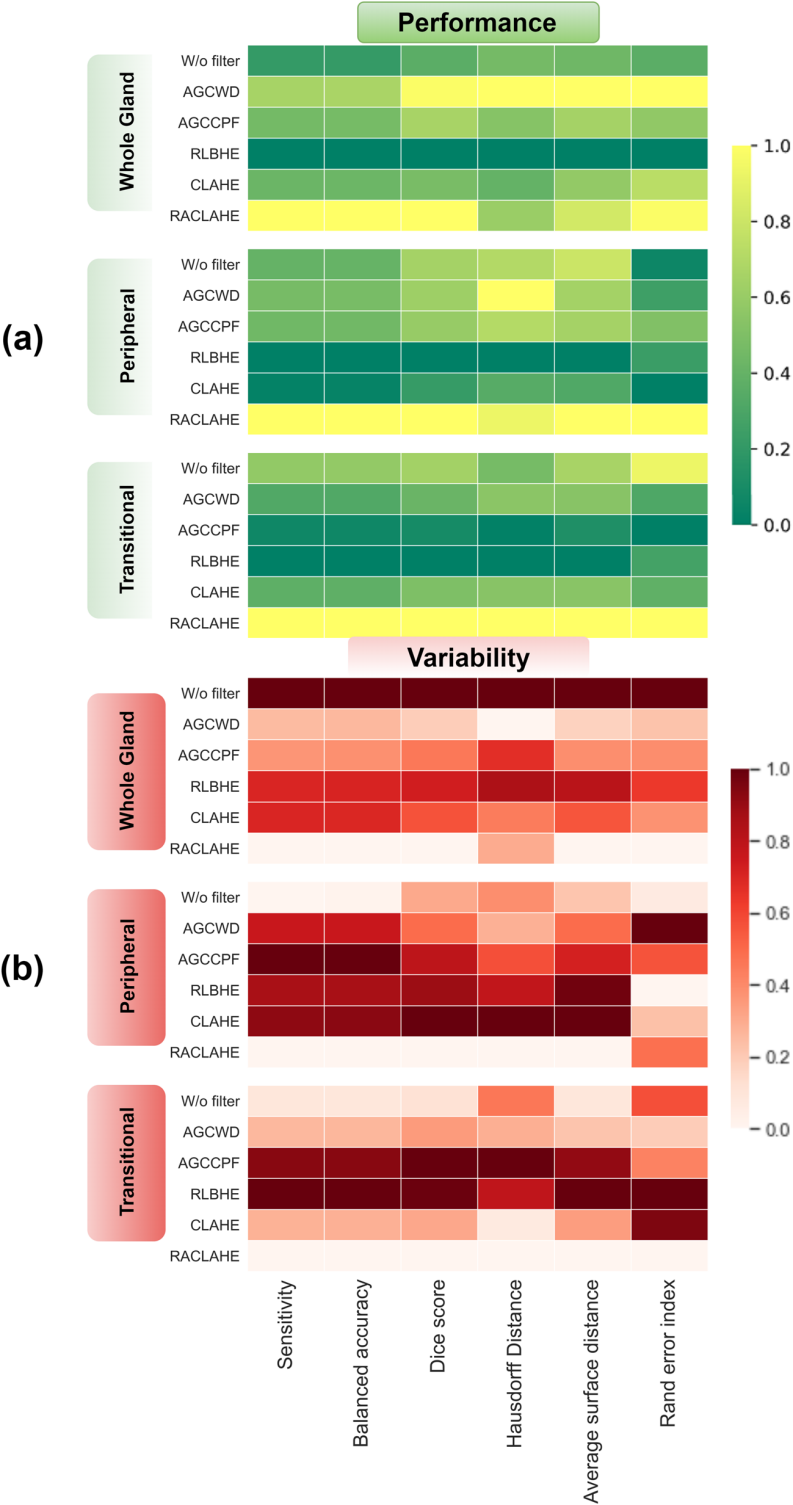
(**a**) Intermodel performance for each metric and histogram based preprocessing technique. The best preprocessing method for each metric is depicted in yellow and the inferior in dark greens. (**b**) Intermodel variability for each metric and the histogram based preprocessing technique. The best method for each metric is depicted in white and the inferior in dark red.

Figure 1a presents the normalized performance for each preprocessing method while Fig. 1b presents the normalized variability across the segmentation models. For a given preprocessing method, the normalization of the results was performed with respect to the minimum and maximum performance for each metric. For instance, in Fig. 1a the best performing preprocessing method in terms of DS has a value of 1 while the worst has a value of 0. Specifically, for the distance-based metrics (HD, ASD) where lower values indicate a better performance, the inverse of these values was computed in order to have a consistency in scale. In Fig. 1b the variability of the mean performance across models, for each preprocessing method and metric, is shown. After normalization, the lowest variability across models is indicated with the value 0 while the highest variability is indicated with the value 1.

Considering Fig. 1a, for WG and PZ segmentation, the RACLAHE outperformed all the other preprocessing methods, in terms of sensitivity, BA, DS and REI, while it had the second-best performance, after AGCWD, in terms of HD and ASD. With respect to variability in performance across DL models, shown in Fig. 1b, the RACLAHE has the lowest standard deviation for WG and PZ segmentation, apart from the HD and REI metric, respectively. Regarding the TZ segmentation, the proposed method was superior for all the metrics with the lowest inter-model variability in performance.

### Explainability of model’s predictions

**Figure 2:**
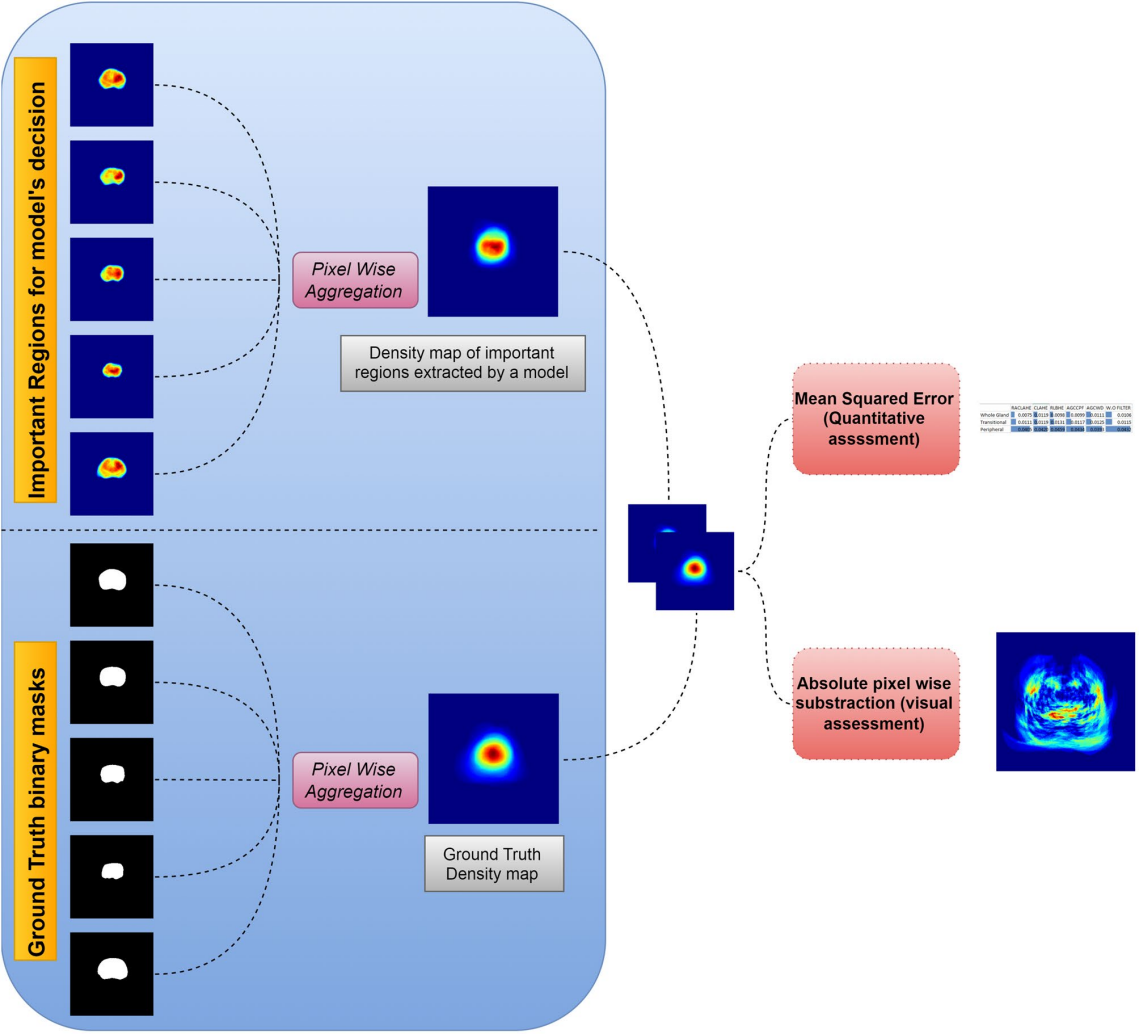
The explainability assessment pipeline. Density maps for GT binary masks and Feature maps are extracted via a pixel wise aggregation. Mean squared error and absolute pixel wise subtraction are performed on the density maps for quantitative and visual inspection.

In order to explain the effect of each preprocessing method on the DL models, we sought to quantify how each model’s important for the task features diverge from the ground truth density maps of the binary masks. Specifically, the density map of ground truth masks are given as:

3 $${GT }_{Density \,map}=\sum_{i,j=0}^{i,j=256}\left\{\sum_{k=0}^{k=Nsl}G{T}_{ij}\left(k\right)\right\},$$

where ${GT }_{Density \,map}$ is the density map that is extracted after the pixel wise aggregation of all binary $GT$ masks, and $G{T}_{ij}(k)$ represents the pixel wise aggregation for the total number of binary ground truth images $Nsl$ in a certain pixel position $i, j$. The density map of important features is given as:

4 $${FM }_{Density \,map}=\sum_{i,j=0}^{i,j=256}\left\{\sum_{k=0}^{k=Nsl}F{M}_{ij}\left(k\right)\right\},$$

where ${FM }_{Density \,map}$ is the density map of extracted features that a model utilize for its decision and is extracted after the pixel wise aggregation of those feature maps, and $F{M}_{ij}(k)$ represents the pixel wise aggregation for the total number $Nsl$ of feature maps extracted by a model in a certain pixel position $i, j$. The latter can take values ranging from 0 to 256 attributed to the spatial dimensions of the density maps. A comprehensive scheme is presented in Fig. 2, while the mean squared error and the absolute subtraction are used as explainability metrics for quantitative and visual assessment. The absolute pixel wise difference map between Eqs. (3) and (4) is given by Eq. (5):

5 $$DMap=\left[\begin{array}{ccc}\left|FM \,\,Density \,\,ma{p }_{\mathrm{0,0}}-GT\,\, Density \,ma{p}_{\mathrm{0,0}}\right|& \cdots & \left|FM\,\, Density \,ma{p}_{\mathrm{0,256}}-GT\,\, Density \,ma{p}_{\mathrm{0,256}}\right| \\ \vdots & \ddots & \vdots \\ \left|FM\,\, Density \,ma{p}_{\mathrm{256,0}}-GT\,\, Density \,ma{p}_{\mathrm{256,0}}\right| & \cdots & \left|FM\,\, Density \,ma{p}_{\mathrm{256,256}}-GT\,\, Density \,ma{p}_{\mathrm{256,256}}\right|\end{array}\right].$$

**Figure 3:**
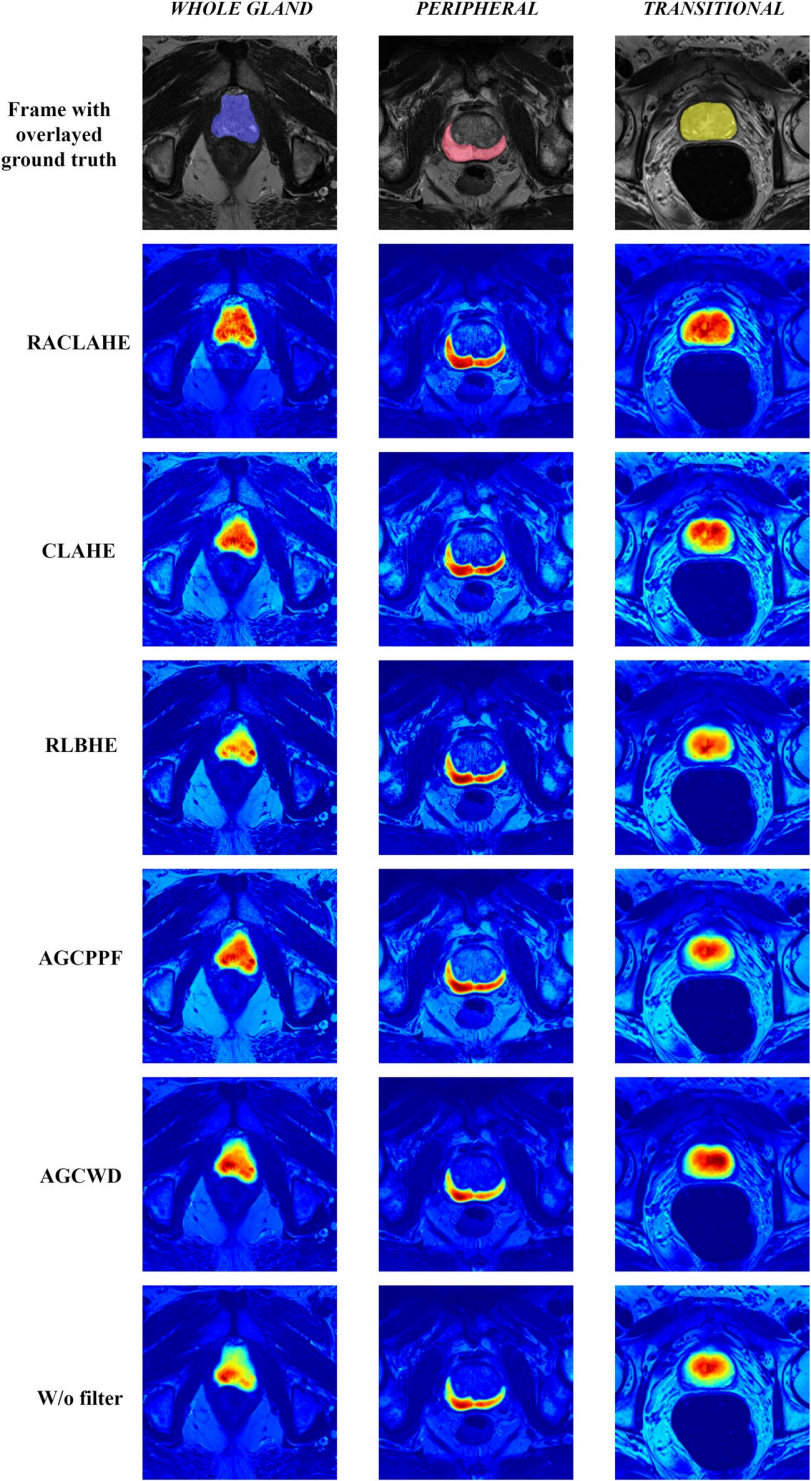
Weighted heatmap for the USE-Net model and for each preprocessing method used. The columns are the prostatic zones while the rows are the evaluated methods.

**Figure 4:**
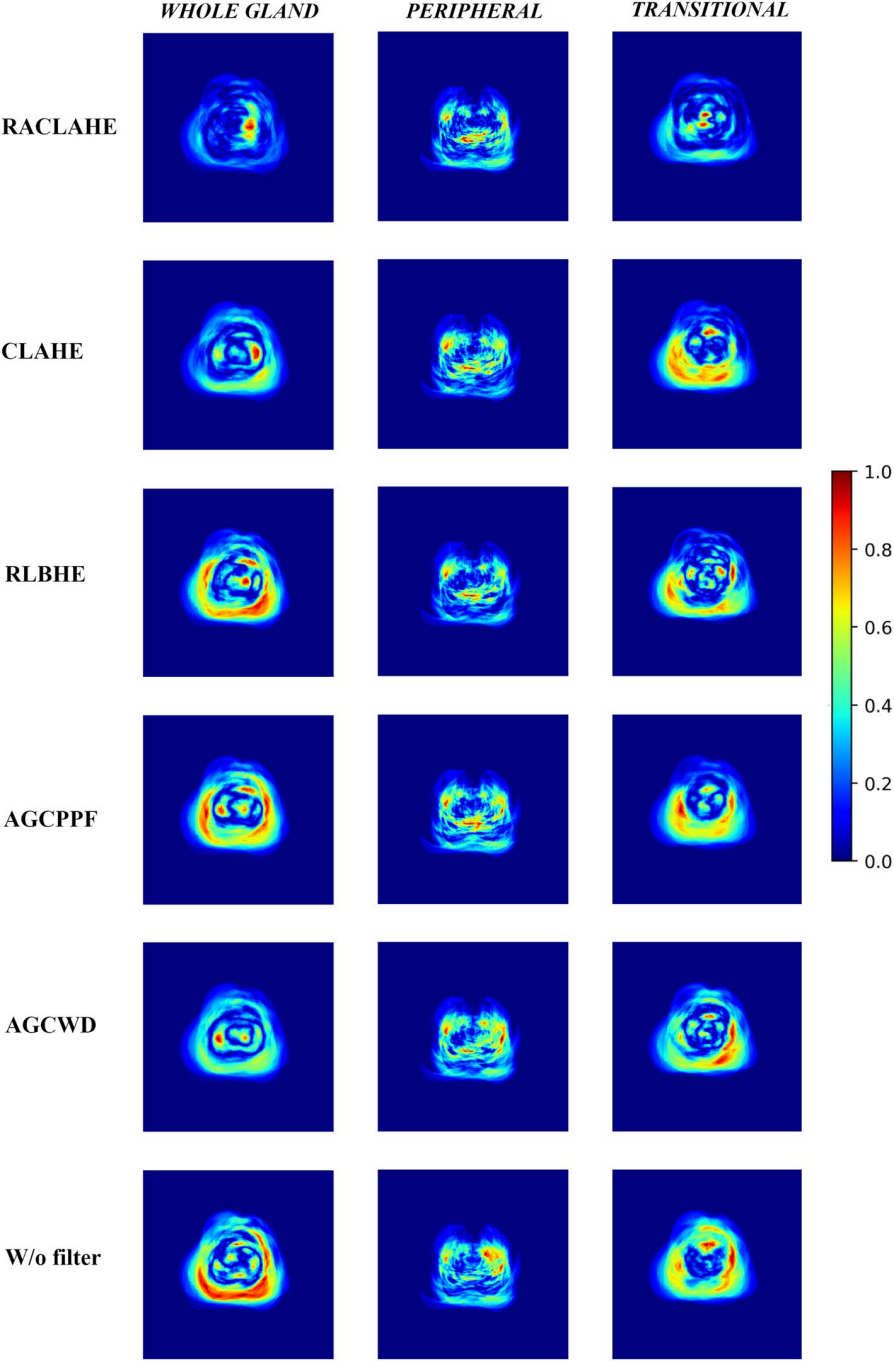
The visual assessment after the absolute pixel wise subtraction of ${GT }_{Density \,map}$ and ${FM }_{Density \,map}$ (Eq. 5), for each preprocessing method applied on the USE-NET network.

The gradient-weighted class activation mapping (Grad-Cam)^37^ technique was used to extract the feature maps (FM) from a certain layer of a DL network. In fact, the performance of a model is tightly linked to the feature maps extracted throughout the forward–backward propagation process. Figure 3 presents the significant features processed by the USE-NET model, under the influence of each preprocessing method, applied for the WG, TZ and PZ segmentation. The RACLAHE method seems to improve the accuracy of boundary estimation assisting the model to focus on relevant pixels. Red areas indicate that the model is certain that those pixels belong to the object of interest, yellow areas imply that the model is less certain for these pixels while blue areas denote that these pixels have no contribution to the model’s final decision. Figure 4 provides a visual representation of the pixel-wise absolute differences (DMap) between the ground truth density map (GT_density map_) and the relevant for the model’s decision features density map (FM_density map_). A near-zero signifies that the pixels significance for a model is equal to its GT density. It is evident that the RACLAHE method has more pixels with values close to the GT, compared to other methods. This suggests that the RACLAHE method can more efficiently assist the DL models in focusing on meaningful features that are showcased by the GT_density map_, reducing their uncertainty.

## Discussion

Despite the widespread usage of image enhancement techniques, they are often adopted on the basis of scant literature evidence and are blindly utilized in a plethora of clinical applications. In this regard, the present work addresses two highly relevant issues in the field of medical image preprocessing, namely, how well image enhancement methods generalize across different segmentation algorithms and how to reliably select the most appropriate preprocessing method for a given task. For the former, we estimated the variability in segmentation performance across models, for each one of the preprocessing methods under evaluation. For the latter, we introduce a feature driven approach to explain models’ predictions, enabling both the qualitative assessment of model performance through visual explanations, as presented in Fig. 4, and the quantitative assessment via the estimation of the absolute mean squared error and the subtraction of feature maps and the GT maps. To the best of our knowledge, this is the first study to evaluate the impact and generalizability of image enhancement methods on DL models for prostate and prostatic zone segmentation.

In contrast to other popular histogram-based image enhancement approaches, which displayed low stability and generalizability across different DL models, the success of RACLAHE lies in the consistent, model-invariant improvement achieved for segmenting the prostate and the prostatic zones. The proposed method’s novelty relies on the combination of an automated DL region proposal model and a local enhancement technique. RACLAHE mainly focuses on (a) the effective automated identification of the region of interest to discretize the relevant for the task region features from the redundant features, (b) the enhancement of the relevant region to enhance the adequate pixels for the segmentation task, and (c) the harmonization of the enhanced region and the redundant region to retransform the image in its original dimensions to be visually presented in a more natural way for the clinical practitioners and the CNNs models as well. Specifically, the RACLAHE was the only technique that did not deteriorate models' performance in any of the experiments conducted, as shown in Tables 1, 2 and 3. In most cases, a superior performance was achieved compared to no-preprocessing or, at worst, the performance remained the same. Regarding the stability of image enhancement methods across different DL models, the RACLAHE was found to be more stable, reducing the variation of results across models and improving the overall inter-model average scores, as shown in Fig. 1.

Another important contribution of this work consists the integration of saliency maps as a performance indicator, providing a unique opportunity to interrogate the effect of different preprocessing methods on models’ behavior. We provide feature map-driven visual explanations to assist on the selection of the most appropriate preprocessing method by highlighting the image features that guide the segmentation task. Herein, the Grad-CAM^37^ technique was employed to visually present class discriminative localization map (heatmap or saliency map) which highlights the most important pixels of a particular class. These heatmaps were coupled with a probabilistic ground truth feature importance map to extract meaningful indications regarding a model’s performance, both qualitatively and quantitatively, as it is shown in Fig. 2. We extend the aforementioned methodology to compare different image enhancement methods and to quantify the contribution of each feature to DL models’ decisions. As depicted in Fig. 4 and Supplementary Table S1, the RACLAHE was able to enhance specific areas within the images that are strongly associated with the ROI. It is worth noting that the most significant differences were observed for the prostate’s WG and the TZ. The proposed method provides better insights about the performance of biomedical imaging applications as it represents a natural way of comparing the ground truth samples from the predicted samples, as indicated in Figs. 2 and 4.

This work has some limitations. Τhe impact of image enhancement was not assessed for 3D segmentation tasks, which would permit to evaluate its effectiveness in every spatial plane. Nonetheless, for this type of analysis, a sufficiently large amount of data is required. Additionally, regarding the RACLAHE method, no hyperparameter tuning was performed to optimally define the cutoff for the CLAHE component and, therefore, the default parameters that are automatically chosen from the algorithm were used. As proposed by Campos et al.^38^, hyperparameter optimization can be achieved by means of machine learning techniques.

In summary, the outcomes of this study indicate that image enhancement using RACLAHE can improve the segmentation efficacy of CNN networks in a model-agnostic manner, thereby, contributing to establishing a concrete image preprocessing pipeline for effective automatic prostate segmentation tasks. Future research may concentrate on establishing the superiority of the proposed method considering different image enhancement techniques and segmentation algorithms and evaluating its generalizability in other population datasets and clinical scenarios. In addition, the application of RACLAHE prior to CNN model training has led to the generation of more accurate saliency maps. These probabilistic pixel-wise representations, reflect a more natural way to visually explain the outcomes of a model. Ultimately, the explainability module will render model’s prediction more trustworthy among clinicals, to further support the decision-making process.

## Methods

### Histogram-based enhancement methods

The four image enhancement techniques used for comparison are described below.

#### Adaptive gamma correction with weighting distribution (AGCWD)

AGCWD is a histogram modification approach to enhance and correct images. The main attribute that differentiates this approach from the power-law transformation is the automatic selection of the gamma factor based on the weighting distribution. Specifically, the authors^39^ used weighting distribution to find the cumulative distribution and therefore specify the gamma parameter. This hyperparameter is set to 1, which is the default value as suggested by the authors in the original work which brightens low pixel intensities on the image and the high intensities remain intact.

#### Adaptive gamma correction with color preserving framework (AGCCPF)

AGCCPF employs a two-step processing approach. First, it improves the contrast and brightness of a given image by modifying the probability distribution of pixels’ intensity and then it applies gamma correction. In the second stage, it restores color using a color-preserving framework. This method is an upgrade of AGCWD due to its ability to retain information better than AGCWD as authors claim^40^. They use a histogram modification function to control the level of contrast enhancement by utilizing the input histogram along with the uniform histogram, which is a histogram equalized image, to produce the resulted one. The difference between input histogram, uniform histogram and the resultant histogram for an image is presented in Fig. S5 in the Supplementary Materials.

#### Range limited Bi-histogram equalization (RLBHE)

RLBHE considers both contrast enhancement and intensity brightness preservation as valuable factors in the output image. First, the single threshold Otsu’s^41^ approach is employed to execute histogram thresholding to get better contrast enhancement and avoid over-enhancement. Second, the range of the equalized input image is limited to ensure that the mean output brightness is almost equal to the mean input brightness, preserving the initial information from the input image and third, each partition of the histogram is equalized independently.

#### Contrast limited adaptive histogram equalization (CLAHE)

CLAHE algorithm^22^ is considered more stable for contrast enhancement operations due to its local application on the frame. This approach is separated into two steps. First the initial image is divided into 8X8 windows that compose the image. Second histogram equalization algorithm is applied to equalize each window independently from the other. In this way, the histogram equalization method does not take into consideration the global features of the image and, therefore, it optimizes the intensity levels to a neighboring area around the center of each window. Compared to the aforementioned techniques, there are several advantages of CLAHE with the main being the reduced contribution of outliers. In histogram equalization, outliers play an important role as the tuning of the histogram is affected by extreme values. With the partition in windows though, extreme values are scaled within a neighborhood region and, therefore, are smoothed.

#### The proposed image enhancement method: RACLAHE

**Figure 5:**
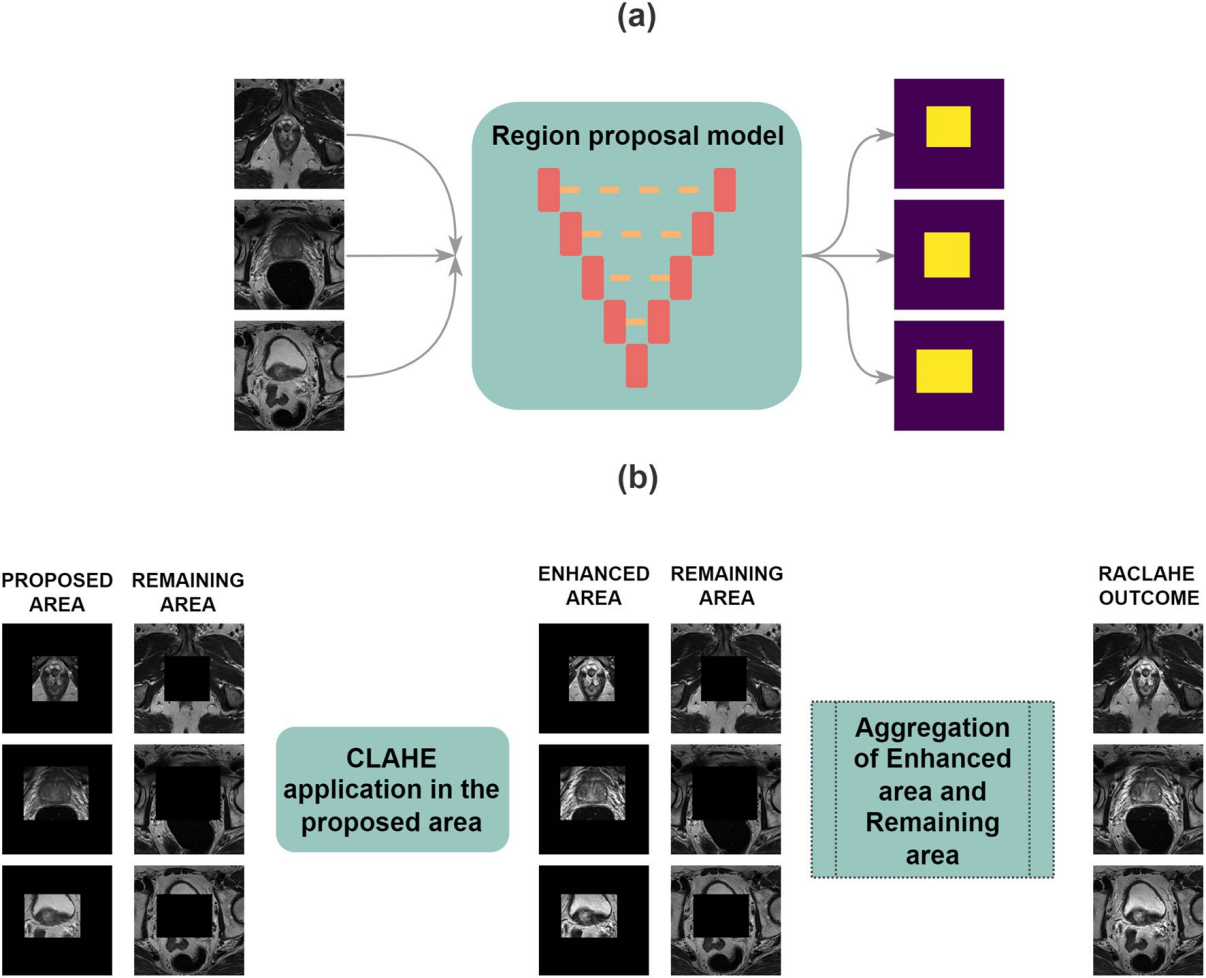
The RACLAHE algorithm. From the initial 256 × 256 frame an area of $\{134\pm 15\} \times \{134\pm 15\}$ pixels is selected which contains the region of interest (**a**). This reduced dimensional space provides more targeted features to be enhanced and simplify the complexity of the problem while introducing some biases to the model regarding the area to identify features from (**b**).

Conventionally, the CLAHE algorithm is applied globally on the entire frame of the image. The algorithm utilizes the histogram equalization method in a close neighborhood around a central pixel. Although the histogram equalization is applied frame-wise, the CLAHE algorithm is applied patch-wise enhancing further the contrast of the sub-regions within the frame. The proposed method RACLAHE utilizes the CLAHE algorithm along with the steps described below to transform selected features to be more interpretable for the model. The pipeline is visually presented in Fig. 5. The algorithm that describes the RACLAHE operation is.

Let $Z$ be a space where the intensity features values of each frame lie, $F{M}_{Z}\in Z, 0\le F{M}_{Z}\le Frame\, width\times Frame\, height$. Each frame is passed from a DL U-Net like structure^9,42^ that proposes a reduced size area that includes the prostate gland. Specifically, the initial space $Z$ is reduced into a subspace $Q\subset Z$ and features $F{M}_{Q}\in Q$ are selected by reducing the dimensionality of the $Z$ space in the $Q$ space. The relation that describes these two spaces is presented in Eq. (6) and the operation is given in Fig. 5a.

6 $$Q\simeq 0.25 Z\pm 0.12 Z.$$

The frame is then divided into two subframes, features $F{M}_{Q}$, $F{M}_{Z}-F{M}_{Q}$ and those areas are the proposed area that contains the whole gland and the remaining area respectively while this process is presented in Fig. 5b. The CLAHE algorithm is then applied on the features $F{M}_{Q}$ (proposed area). Specifically, $F{M}_{Q}$ pixel intensity features are divided into $8\times 8$ patches and the number of those patches in each $F{M}_{Q}$ is approximately 196. Then, the probability of the occurrence of each pixel’s unique intensity value ${P}^{patch}({i}_{F{M}_{Q}})$ is given as:

7 $${P}^{patch}\left({i}_{F{M}_{Q}}\right)=\frac{Num\left({i}_{F{M}_{Q}}\right)}{TotNum},0\le {i}_{F{M}_{Q}}\le L{D}^{patch},$$

where $Num({i}_{F{M}_{Q}})$ is the number of occurrences of pixel intensity ${i}_{F{M}_{Q}}$ within the patch, $TotNum$ is the total number of pixels of the patch, $L{D}^{patch}$ is the range of values, inside each patch. Consequently, the cumulative distribution for each patch is calculated:

8 $$CD{F}^{patch}({i}_{F{M}_{Q}})=\sum\limits _{k=0}^{{i}_{FMQ}}{P}^{patch}\left(k={i}_{F{M}_{Q}}\right).$$

The histogram equalized patch is obtained by Eq. (9) making use of Eqs. (8) and (7):

9 $$EqHis{t}^{patch}=round\left(L{D}^{patch}-1\right)\times {CDF}^{patch}\left({i}_{F{M}_{Q}}\right).$$

The enhanced area of Fig. 5b is constructed from the aggregation of the histogram equalized patches and it is obtained as:

10 $$F{{M}_{Q}}^{trans}=Enhanced\, Area=\sum \limits_{t=0}^{Patches}EqHis{t}^{t} ,$$

where with $Patches$ we denote the total number of patches within $F{M}_{Q}$ while $F{{M}_{Q}}^{trans}$ indicates the enhanced area. Finally, the RACLAHE resulted image is given by Eq. (11) it is shown in Fig. 5b:

11 $$RACLAHE =F{{M}_{Q}}^{trans}+F{M}_{Z}-F{M}_{Q}.$$

### Model development

Five CNN algorithms were implemented to evaluate the impact of preprocessing methods to segment the prostate and the prostatic zones, namely the U-Net^9^, ResUNet^43^, U-Net3+^44^, U-Net++^45^ and USE-NET^46^ while a brief description of them is given in the Supplementary Materials. For model training, the Prostate X dataset was split into the training and the validation sets where the 85% of image frames were used for training and the remaining 15% for validation. The splits were kept the same for all the experiments run in this study (i.e. for the different models and preprocessing methods). The initial learning rate was kept at 0.0001 whereas the batch size and epochs were 16 and 120, respectively, while the adam optimizer was used for weight updating throughout the training process. As loss function the sigmoid focal crossentropy^47^ was utilized due to its effectiveness to handle unbalanced data. The early stopping technique used in order to stop model training when the validation performance stopped improving further. The segmentation performance of each model and the preprocessing method was evaluated externally on an independent dataset (Prostate 3 T). The GPU used for the experiments is the NVIDIA Quadro P6000, the drivers are of version 441.66 while the python packages used are numpy = 1.21.2, keras-unet-collection = 0.1.11, scikit-image = 0.18.3, SciPy = 1.7.1, Tensorflow = 2.2.0 and Tensorflow-addons = 0.11.2. The original code and the docker image for RACLAHE and all the experiments are available from the authors upon request.

### Performance assessment

Several metrics have been implemented to thoroughly evaluate the performance of the proposed method and existing histogram modification methods. Specifically, DS, REI, Sensitivity, BA, HD and ASD common segmentation metrics were computed thanks to the complementary information they provide which could provide sufficient insights about models’ performance. DS and REI are metrics related to the overlapping between the predicted and the true annotation of the object of interest. On the other hand, Sensitivity and BA provide information about the ability of the model to identify an area of interest with high class imbalance between the background and foreground pixels. HD and ASD employ one dimensional measurement that connect the relevant results with real world insights (S.I unit system). Specifically, HD and ASD are measurements of how far two data points are, one belonging to the ground truth boundary and the other to the prediction. Herein, the 95% HD was employed to avoid using extreme values as they may not be indicative of real model performance. All the performance metrics were computed on the external testing dataset. The Wilcoxon rank-sum test (two-sided) was used to compare the proposed RACLAHE technique with all the other methods and a p-value ≤ 0.05 was considered as significant in performance differences.

## Supplementary Information

Supplementary Information.

## Data Availability

The datasets generated during and analyzed during the current study are available in the TCIA repository and in GitHub. Specifically the Prostate X2 dataset can be found in this link (https://github.com/rcuocolo/PROSTATEx_masks) while the Prostate 3T dataset can be found in this link (https://wiki.cancerimagingarchive.net/display/Public/Prostate-3T). Those are publicly available datasets. Docker container of the RACLAHE method and code is available from the authors upon request.
