# Supplementary material for: Region-adaptive magnetic resonance image enhancement for improving CNN-based segmentation of the prostate and prostatic zones
Source: Sci Rep. 2023 Jan 13;13:714. doi: 10.1038/s41598-023-27671-8 (PMC9837765; doi:10.1038/s41598-023-27671-8)
Supplement: Supplementary file 1 — Supplementary Information. [file 41598_2023_27671_MOESM1_ESM.pdf]

Supplementary Information

Region-Adaptive Magnetic Resonance Image Enhancement for improving CNN-based segmentation of the prostate and prostatic zones

Dimitrios I. Zaridis[1], Eugenia Mylona[1,+], Nikolaos Tachos[1] ,Vasileios C. Pezoulas[2], Grigorios Grigoriadis[2], Nikos Tsiknakis[3], Kostas Marias[3], Manolis Tsiknakis[4], Dimitrios I. Fotiadis[1,2,*]

[1]Department of Biomedical Research, Institute of Biomedical Research, Foundation for Research and Technology – Hellas (FORTH), Ioannina, Greece

[2]Unit of Medical Technology and Intelligent Information Systems, Department of Materials Science and Engineering, University of Ioannina, Ioannina, Greece

[3]Institute of Computer Science, Foundation for Research and Technology – Hellas (FORTH), Heraklion, Greece

[4]Department of Electrical Computer Engineering, Hellenic Mediterranean University, Heraklion, Greece

Co-corresponding authors:

*Dimitrios I. Fotiadis (fotiadis@uoi.gr)

Dataset analysis

In order to better examine the aforementioned prostatic areas, a descriptive analysis was conducted to quantify the inter- and intra-patient volume variations of the different prostatic regions. As it is shown in Fig. S1A, within a single patient, the base, the midgland and the apex, corresponding to the first, middle and last axial frames of the prostate, respectively, have different sizes, with the prostate's midgland being much larger than base and apex. This renders the automatic segmentation of prostatic zones particularly challenging, since the algorithm has to distinguish different areas with amorphous shape and dimensions. On top of that, the prostate is characterized with high inter-patient variability, as demonstrated in Fig. S1B, which is particularly evident for the midgland region with values ranging from 15 cm^3 up to 100 cm^3 (100ml). This is due to the fact that often prostate cancer patients suffer from conditions that lead to an increase of the prostate's volume such as prostatitis or hyperplasia. This variability adds to the complexity of automating the segmentation process.

Deep learning segmentation networks

For the evaluation of the image preprocessing techniques investigated in this paper, we selected and implemented five DL models from the family of encoder-decoder networks which have demonstrated state-of-the-art performance in segmentation tasks.

Unet

U-Net architecture is an encoder-decoder architecture which serves two purposes: i) dimensionality reduction, and ii) precise localization of the object of interest. Each block of the encoder network is comprised of a convolutional layer to extract feature maps from a batch normalization layer to normalize each batch's feature maps, an activation function layer which enables certain neurons to propagate their information forward while it shuts several other neurons that do not contribute in the given task, and a max pooling layer to reduce the dimensionality of the input. On the other hand each decoder block is similar to those of the encoder with the only difference being the transposed convolutional layers which reduces the feature maps condensing the information and the upsampling layers that are opposite to the max pooling layers increasing the dimensions of the given input.

ResUnet

The architecture of ResU-Net is conceptually similar to the U-Net with the difference of the addition of residual connections. It consists of an encoder, to dimensionally reduce the image and identify patterns related to the object of interest and a decoder, which reconstructs the image in its original dimensions and identifies patterns about the position of the object of

interest. Residual connections assist the ResU-Net model with interactions between its layers and halves the computational time and load of the network. Residual connections transfer the aggregation of information originated by the input and the information after the operation within the block, ensuring in this way that features will not lose information from the initial input image.

Unet++

The U-Net++ is a successor to the original U-net encoder-decoder model. The ensembled networks inside the outer encoder-decoder is the main characteristic of this model. Specifically, U-Net++ contains three encoder-decoder networks, with each network being more capable of extracting complicated features than the one before it. To construct the final ensembled forecast, the outputs from each separate encoder-decoder network are joined. This is called deep supervision and it contributes to the output of swallow features along with deeply extracted ones. Therefore the network is capable of identifying simple patterns as well as complicated ones.

Unet3+

The main attribute of the U-Net 3+ architecture is that each block of the encoder path is interconnected with each block of the decoder path. In this manner, the model is capable of transferring the information retrieved by the convolutional layers of each block of the encoder and combining it on each block of the decoder by automatically configuring the dimensions requirements. This approach ensures that information flow is increased and the network works at a full scale level. The full-scale deep supervision is used in order to develop hierarchical representations from full-scale aggregated feature maps. In contrast to U-Net++'s deep supervision of the created full-resolution feature map, U-Net 3+ generates a side output from each decoder step that is supervised by the ground truth.

USE-NET

The main attributes of the USE-NET network that differentiate its operations from other similar networks are the squeeze and excitation blocks. This layer incorporates a distinct component of architectural design - the channel relationship- which is capable of increasing a network's representation capability by explicitly modeling the interdependencies between its convolutional features' channels. A feature recalibration technique that allows the network to learn to use global information is utilized, to selectively emphasize informative characteristics while suppressing less helpful ones. While the convolutional operations extract intrachannel features the squeeze and excitation blocks are structured in a way that allows to correlate interchannel features providing increased intercommunication between model’s blocks and assisting in the extraction of more meaningful features related to the task.

Adaptive Gamma Correction with Weighting Distribution (AGCWD)

AGCWD is obtained through Equations (1), (2), (3) and (4) describes the power transformation law applied on images:

$$PTL(h) = h_{max}(h/h_{max})^{\gamma} \tag{1}$$

where $PTL(h)$ is the transformed pixel with intensity h, h_{max} is the maximum intensity level and γ is the gamma parameter to adjust. The weighting distribution function is given by equation (2),

$$PDF_{wgt}(h) = PDF_{max}(\frac{PDF(h) - PDF_{min}}{PDF_{max} - PDF_{min}})^{a} \tag{2}$$

where $PDF_{wgt}(h)$ is the weighted probability distribution function of intensity level h, $PDF(h)$ is the probability distribution function of intensity level h and a is a hyperparameter which normally takes value 1 as suggested by the authors on the original work and it is the default value. Then the weighted cumulative distribution function is calculated through equation (3).

$$CDF_{wgt}(h) = \sum_{h=0}^{h_{max}} \frac{PDF_{wgt}(h)}{\sum PDF_{wgt}} \tag{3}$$

where $CDF_{wgt}(h)$ is the weighted cumulative distribution of intensity level h, $PDF_{wgt}(h)$ is the weighted probability distribution function of intensity level h and $\sum PDF_{wgt} = \sum_{h=0}^{h_{max}} PDF_{wgt}(h)$. The output gamma parameter is defined by equation (4) through equation (3)

$$\gamma = 1 - CDF_{wgt}(h) \tag{4}$$

Adaptive Gamma Correction with Color Preserving Framework (AGCCPF)

Their main concept of AGCCPF is described in equation (10).

$$MH = c\ Hist + (1 - c)UniHist, 0 \leq c \leq 1 \tag{5}$$

where MH is the modified histogram, $Hist$ is the histogram of the input, $UniHist$ is the uniform histogram of the input. Equation (5) is an optimization problem where c is a parameter which needs to be identified by keeping MH similar to $Hist$ and the difference between MH and $UniHist$ to be as minimal as possible.

Range Limited Bi-Histogram Equalization (RLBHE)

In order to produce the processed image after the RLBHE, the authors[43] employ the absolute mean brightness error (AMBE) and they try to minimize it through equation (6).

$$(B'_{upper}, B'_{lower}) = argmin_{B'_{upper}, B'_{lower}}\{(aB'_{lower}B'_{upper} - b)^2\} \tag{6}$$

$$0 \leq B'_{lower} \leq TH_o\ , TH_o \leq B'_{upper} \leq B_{upper}$$

Where B'_{upper}, B'_{lower} are the boundaries that minimize the AMBE, $a = \sum_{i=0}^{TH_O} P(h_i)$ and $P(h_i)$ is the probability of occurrence of the intensity h_i, TH_O is the single threshold proposed by Otsu's method, $b = 2h_{mean} - h_{TH_O} - [1 - \sum_{i=0}^{TH_O} P(h_i)]$, h_{mean} is the mean intensity and h_{TH_O} is the intensity at the threshold proposed by Otsu's method.

Figures

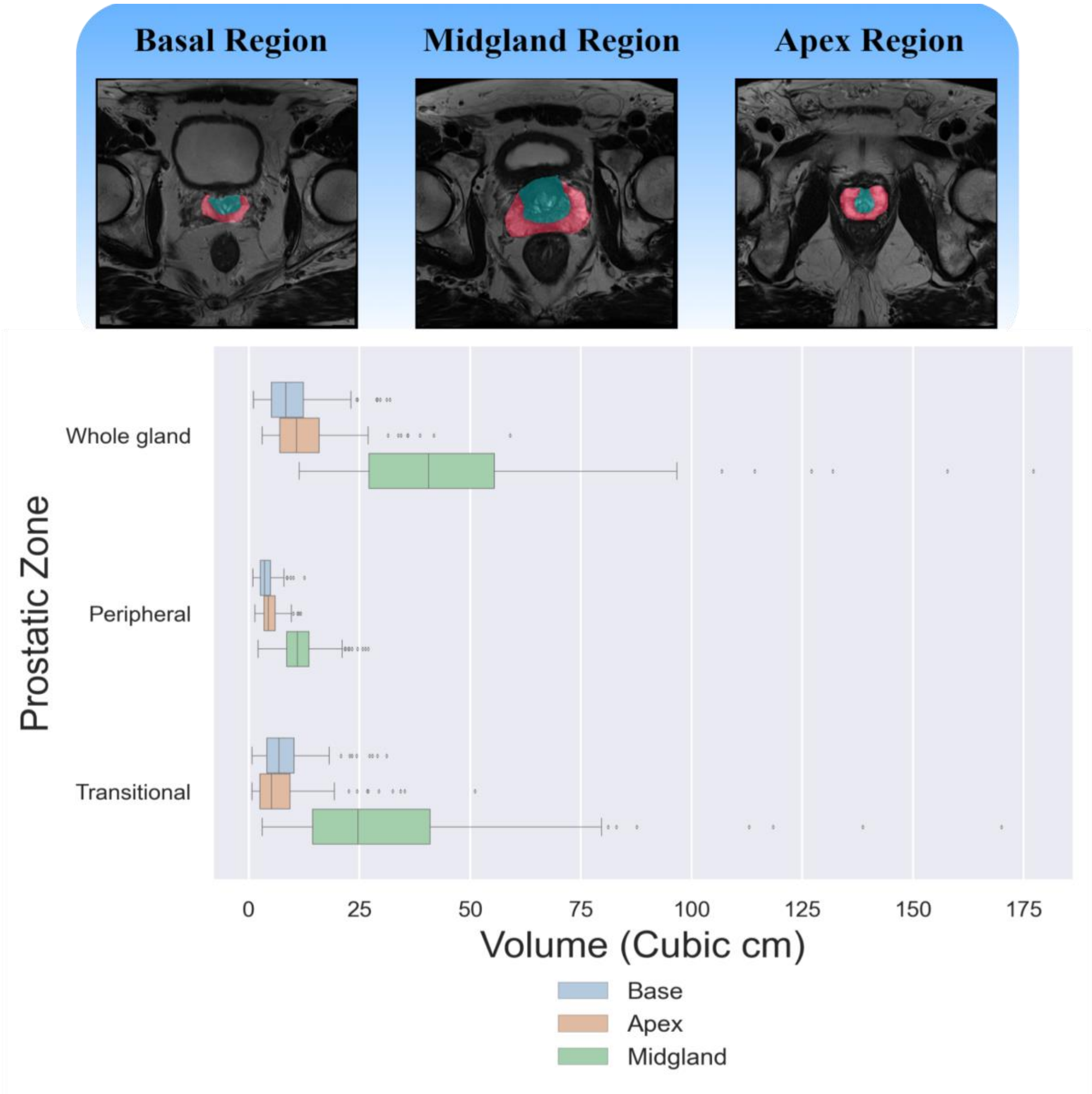

Figure S1. Intra- and inter-patient prostate size variations: (A) The basal (left), midgland (middle) and apex (right) axial MRI slices of the prostate with the corresponding annotations of PZ (red) and TZ (blue); (B) Boxplots of WG, PZ and TZ size in the training population for the base, the midgland and the apex.

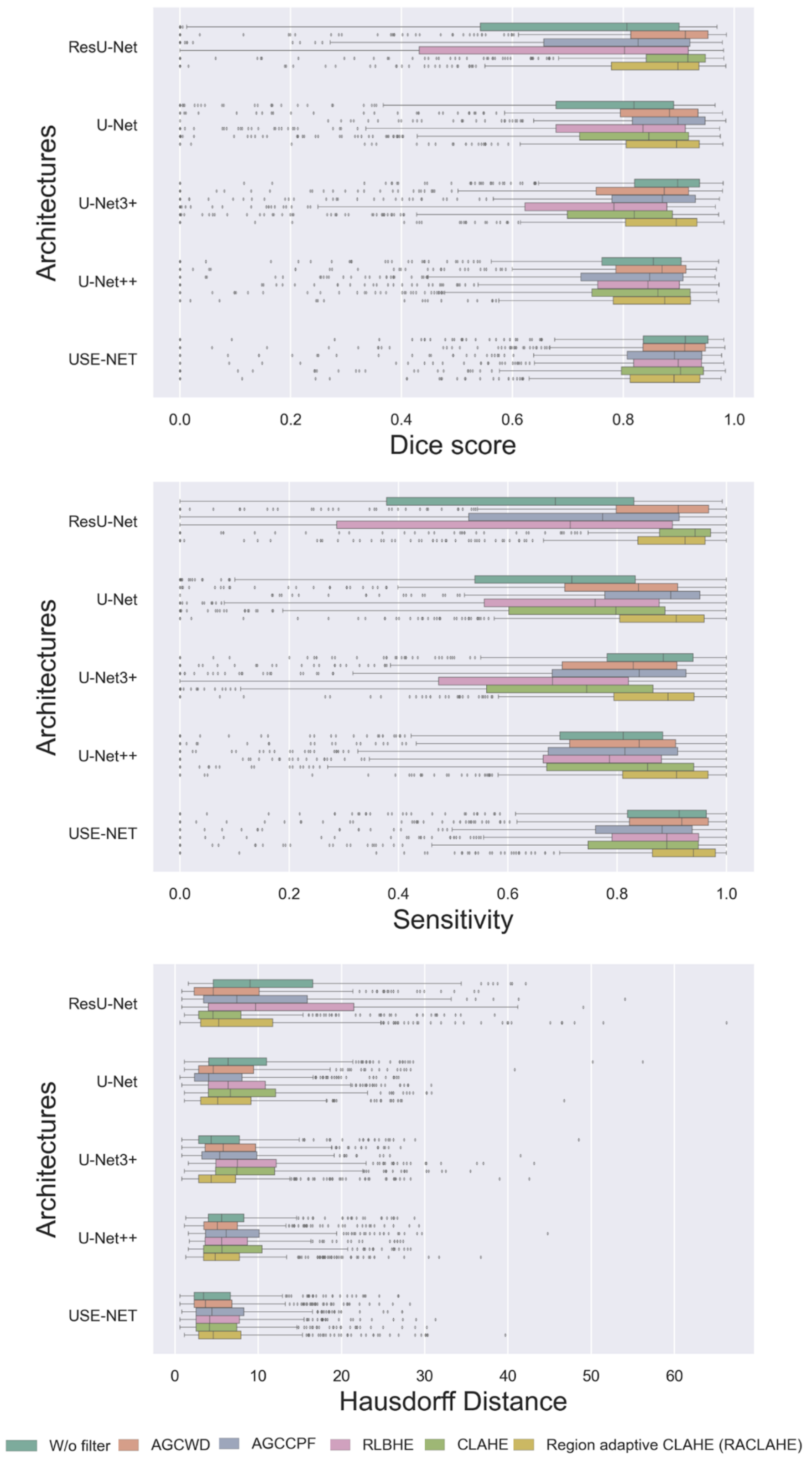

Figure S2. Boxplots of WG segmentation performance for each model and preprocessing techniques.

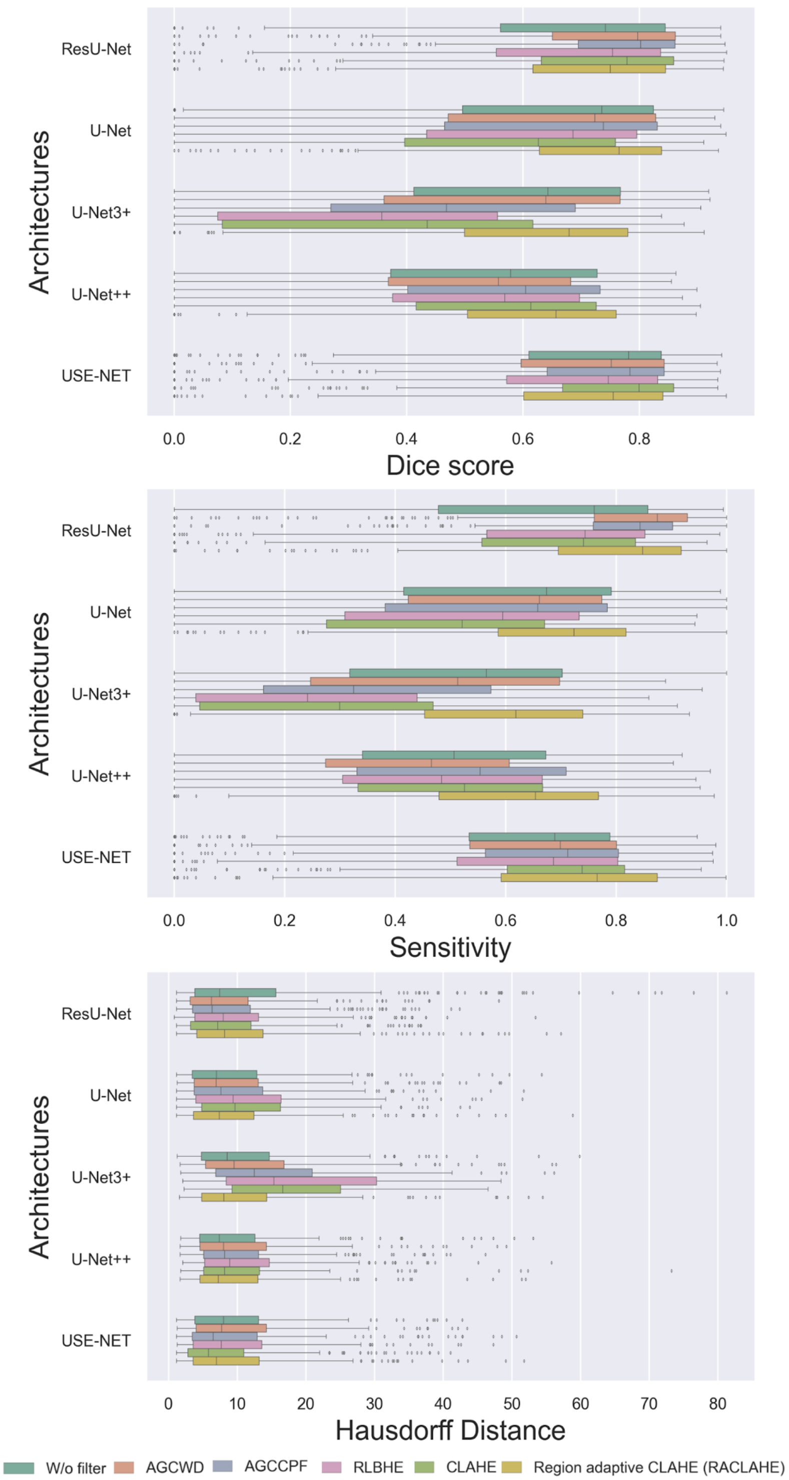

Figure S3. Boxplots of PZ segmentation performance for each model and preprocessing techniques.

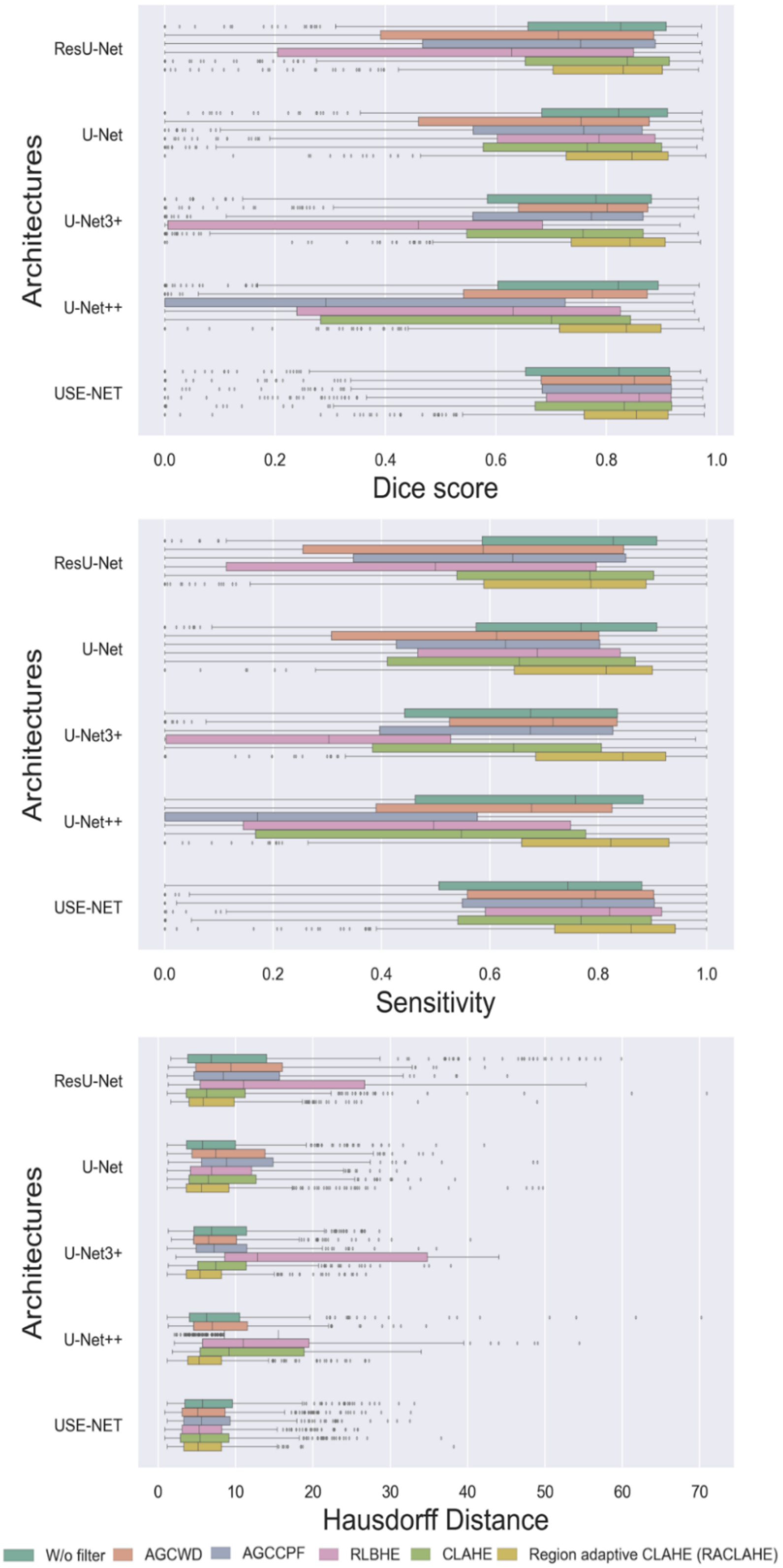

Figure S4. Boxplots of TZ segmentation performance for each model and preprocessing techniques.

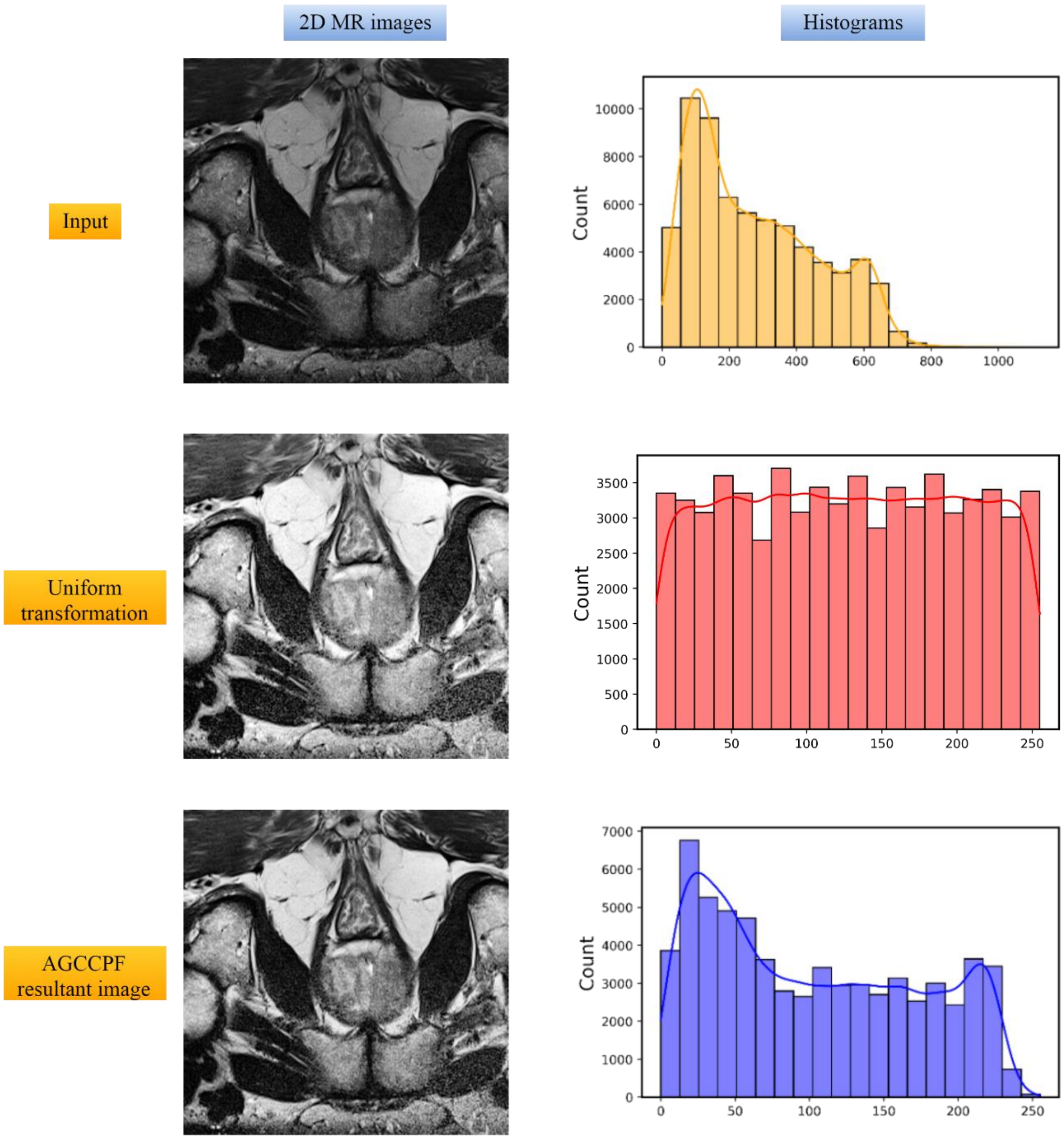

Figure S5. Example of AGCCPF process in a single image. Initial image, uniformed image and resultant image along with their corresponding histograms.

Tables

Table S1. Mean Squared error for each filter in USE-NET model regarding the GT density maps and the Feature maps density maps.

	W/o FILTER	AGCWD	AGCCPF	RLBHE	CLAHE	RACLAHE
Whole Gland	0.0008	0.0011	0.0014	0.0009	0.0009	**0.0004**
Peripheral	0.0031	0.0033	0.0027	0.0025	0.0023	**0.0019**
Transitional	0.0014	0.0007	0.0016	0.0005	0.0011	**0.0004**
